# Application of polymerized porcine hemoglobin in the *ex vivo* normothermic machine perfusion of rat livers

**DOI:** 10.3389/fbioe.2022.1072950

**Published:** 2022-12-01

**Authors:** Bin Li, Jie Zhang, Chuanyan Shen, Tingting Zong, Cong Zhao, Yumin Zhao, Yunhua Lu, Siyue Sun, Hongli Zhu

**Affiliations:** ^1^ The College of Life Sciences, Northwest University, Xi’an, Shaanxi, China; ^2^ National Engineering Research Center for Miniaturized Detection Systems, Northwest University, Xi’an, China; ^3^ Key Laboratory of Resource Biology and Biotechnology in Western China, Ministry of Education, School of Medicine, Northwest University, Xi’an, China

**Keywords:** organ preservation, liver transplantation, normothermic mechanical perfusion, pPolyHb, liver injury indexs

## Abstract

**Background:** In contrast to traditional static cold preservation of donor livers, normothermic machine perfusion (NMP) may reduce preservation injury, improve graft viability and potentially allows *ex vivo* assessment of graft viability before transplantation. The polymerized porcine hemoglobin is a kind of hemoglobin oxygen carrier prepared by crosslinking porcine hemoglobin by glutaraldehyde to form a polymer. The pPolyHb has been proved to have the ability of transporting oxygen which could repair the organ ischemia-reperfusion injury in rats.

**Objective:** In order to evaluate the effectiveness of rat liver perfusion *in vitro* based on pPolyHb, we established the NMP system, optimized the perfusate basic formula and explored the optimal proportion of pPolyHb and basal perfusate.

**Methods:** The liver was removed and perfused for 6 h at 37°C. We compared the efficacy of liver perfusion with different ratios of pPolyHb. Subsequently, compared the perfusion effect using Krebs Henseleit solution and pPolyHb perfusate of the optimal proportion, and compared with the liver preserved with UW solution. At 0 h, 1 h, 3 h and 6 h after perfusion, appropriate samples were collected for blood gas analysis and liver injury indexes detection. Some tissue samples were collected for H&E staining and TUNEL staining to observe the morphology and detect the apoptosis rate of liver cells. And we used Western Blot test to detect the expression of Bcl-2 and Bax in the tissues.

**Results:** According to the final results, the optimal addition ratio of pPolyHb was 24%. By comparing the values of Bcl-2/Bax, the apoptosis rate of pPolyHb group was significantly reduced. Under this ratio, the results of H&E staining and TUNEL staining showed that the liver morphology was well preserved without additional signs of hepatocyte ischemia, biliary tract injury, or hepatic sinusoid injury, and hepatocyte apoptosis was relatively mild.

**Conclusion:** Through the above-mentioned study we show that within 6 h of perfusion based on pPolyHb, liver physiological and biochemical activities may essentially be maintained *in vitro*. This study demonstrates that a pPolyHb-based perfusate is feasible for NMP of rat livers. This opens up a prospect for further research on NMP.

## Introduction

Liver transplantation is the primary form of therapy for end-stage liver disease. The success rate and long-term survival rate of liver transplantation have significantly increased thanks to improvements in surgical technique, perioperative care, and the use of immunosuppressants. Although liver transplantation has successfully resolved the therapeutic conundrum for patients with liver illnesses, it has also brought new issues, such as the limited availability of transplantation owing to a shortage of organ donors. The number of people who require liver transplantation is far higher than the number of eligible liver donors, despite the fact that treatments for liver disorders are continually being improved. The increased use of extended standard donors has been encouraged by the demand for liver transplantation donors (Tullius and Rabb, 2018). The use of extended standard donors for liver transplantation, however, increases the risk of postoperative problems, such as early allograft dysfunction (EAD), primary non-functionality, or severe chronic sequelae. The liver from both donation after brain death (DBD)and donation after cardiac death (DCD) donors may sustain long-term thermal ischemia damage or a hypotension attack (Vogel et al., 2012). The optimization study on some marginal liver donors and elderly liver donors has also been more in-depth in recent years.

The most used technique for *in vitro* organ preservation in clinical practice is static cold storage (SCS) (Jing et al., 2018). The absence of metabolic substrates and the buildup of metabolites would nonetheless harm the donor liver since the low temperature (0–4°C) environment slows down but does not entirely cease organ metabolism (Clavien et al., 1992; Jaeschke, 1998). Normothermic machine perfusion of liver (NMP-L) is a technique for preserving the graft under near-physiological conditions (Vogel et al., 2017). The liver is quickly stripped out by vascular cannulation and removal of excess tissue, it is then connected to a heparinized circuit filled with warm, oxygenated blood and supplied with nutrients. Compared with static cold storage at low temperature, this technology is more in line with physiological characteristics. In contrast to SCS, Normothermic machine perfusion (NMP) is dynamic and it provides a continuous supply of oxygen and other metabolic precursors at physiological temperature. Liver transplantation studies in pigs have shown that NMP-preserved grafts, including those with significant warm ischemia injury before transplantation, which with good post-reperfusion function and survival compared with grafts preserved with SCS (Schön et al., 2001; Brockmann et al., 2009; Fondevila et al., 2011). In human clinical studies, NMP has been shown to lead to glycogen recruitment, which stores graft energy (Mergental et al., 2016). Given that the liver is fully metabolically active, NMP also provides the best opportunity to assess graft viability prior to reperfusion *in vivo* (Perk et al., 2011). The perfusate used for liver NMP usually consists of crystalline or colloidal solutions, oxygen carriers, calcium, broad-spectrum antibiotics, insulin, and heparin (Butler et al., 2002; op den Dries et al., 2013; Vogel et al., 2017; Liu et al., 2018). Depending on the duration of perfusion, metabolic substrates, including glucose or parenteral nutrition, trace elements and vitamins, may also be added. Given the high metabolic demands at 35–37°C, NMP requires the use of oxygenators and the addition of oxygen transporters to the perfusate to use sufficient oxygen carriers to deliver oxygen throughout the organs during perfusion. Red blood cell (RBC) is the most commonly used oxygen carrier for NMP in clinical practice, but it is easy to cause hemolysis due to red blood cell rupture during perfusion (Laing et al., 2017). Currently, hemoglobin-based cell-free solutions are also used for hepatic NMP (Fontes et al., 2015; Laing et al., 2017). In addition, non-cellular hemoglobin oxygen carriers such as HBOC-201 (Hemopure, HbO_2_ Therapeutics LCC, Souderton, Pennsylvania, USA) can provide oxygen dissociation properties similar to those of red cell hemoglobin, allowing the use of NMP without changing the perfusate (de Vries et al., 2019).

Hemoglobin-based oxygen carriers (HBOCs) may now be mechanically perfused *in vitro* at room temperature, according to research. Using HBOCs to preserve organs *in vitro* may have the following potential advantages: effective preservation of cell metabolism and reduction of hypoxia-induced damage, the diameter is significantly smaller than that of red blood cells to more effectively carry oxygen, without immunological response, lengthy storage time and good stability, broad tolerance for temperature, readily available from a variety of sources (Cao et al., 2021). In this study, polymerized porcine hemoglobin (pPolyHb) was used as the oxygen carrier during NMP perfusion. Pig hemoglobin (pHb), which serves as the raw material, is chemically changed to form a novel type of polymeric hemoglobin oxygen carrier called polymerized porcine hemoglobin (pPolyHb) (Zhang et al., 2012). pPolyHb possesses the usual properties of HBOCs.Furthermore, the raw material pHb is extremely similar to human hemoglobin (hHb), has abundant resources (Zhu et al., 2011a). In the previous study, our laboratory conducted systemic exchange transfusion and emergency transfusion of hemorrhagic shock animals. From the aspects of hemodynamics, acid-base balance metabolism, and oxygen delivery, pPolyHb has good capacity of expansion, oxygen carrying and release, and can be used for normal tissue perfusion and hemodynamic stability in experimental animals, reaching the standard of replacing red blood cells. In this study, we constructed a normothermic machine perfusion system for isolated rat liver, designed the perfusate based on pPolyHb which as oxygen carrier for the first time, in order to explored the optimal proportion of pPolyHb in the perfusate as oxygen carrier, and evaluated its effectiveness in the perfusion of isolated rat liver, thereby demonstrating the utility of liver preservation.

## Materials

### Animals

Male Sprague-Dawley rats weighing 240 ± 20 g were used in the study (n = 42). The animals were kept in standard temperature conditions (22 ± 1°C), relative humidity of 55%–58%, 12 h/12 h light and dark cycle, with free access to food and water. The experiments described in this study were performed in accordance with the guidelines of the National Institutes of Health on the use of experimental animals. Approval of the Animal Care Committee of Northwest University was obtained prior to initiating the experiments.

### Solution used in the experiment

pPolyHb: 10.5 ± 0.5 g/dl pPolyHb, methemoglobin <5%, endotoxin <1.0 EU/mL, osmolality 300–330 mOsm, pH 7.4 ± 0.05, average molecular weight of pPolyHb 600 ± 50 kDa, 64 kDa tetramer <2%. Prepared in buffers composed of Na^+^ 135–155 mmol/L, K^+^ 3.0–5.0 mmol/L, Ca^2+^1–3 mmol/L, and Cl^−^ 140–160 mmol/L, and stored in nitrogen at 4°C until use (Zhu et al., 2011b). Briefly, hemoglobin from fresh porcine blood was purified through specific steps, and then cross-linked by glutaraldehyde. Small molecules, including excess glutaraldehyde and tetramer-hemoglobin, were removed by ultrafiltration. Additional details regarding pPolyHb are currently being withheld because of pending patents. The P50 of pPolyHb is 20–28 mmHg.

Basal perfusate: Hydroxyethyl starch, 0.9% NaCl injection, MgSO_4_, CaCl_2_, KH_2_PO_4_, NaHCO_3_, Compound Amino Acid Injection, Glucose, Insulin, L-glutamine, Penicillin, Dexamethasone, Heparin.

Krebs-Henseleit solution: Potassium 5 mmol/L, Sodium 150 mmol/L, Calcium 2 mmol/L, Magnesium 1.2 mmol/L, Chloride 131 mmol/L, Phosphate 1.25 mmol/L, Sulfate 1.2 mmol/L, Glucose 10 mmol/L, Osmolality 312 mOsm/L (Chien et al., 2000).

UW solution: Potassium 125 mmol/L, Sodium 30 mmol/L, Magnesium 5 mmol/L, Phosphate 25 mmol/L, Sulfate 5 mmol/L, Glutathione 3 mmol/L, Adenosine 5 mmol/L, Lactobionate 100 mmol/L, Raffinose 30 mmol/L, Allopurino l1 mmol/L, Dexamethasone 20.3 mmol/L, Hydroxyethyl starch 5 g/dl, Insulin 40 U/L, Osmolality 320 mOsm/L (Chien et al., 2000).

### Perfusion system

**FIGURE 1:**
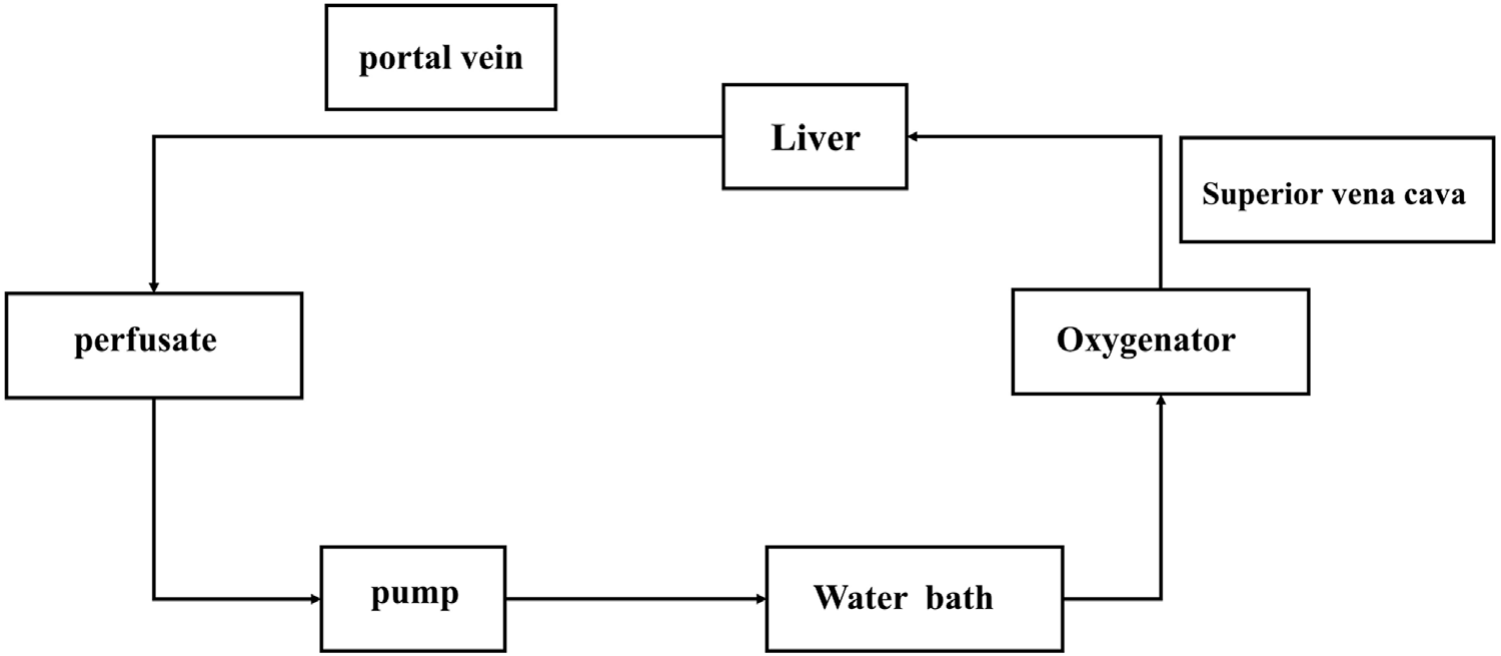
Normothermic machine perfusion system. The pump is the main switch of the system. After the corresponding parameters are set, the filling liquid starts to flow. After the perfusate flows through the pump (if has bubbles, they will be displayed here, and the perfusion will be stopped immediately), it will connect to the blood inlet end of the oxygenator, the bleeding end will be connected to the portal vein of the liver, and the perfusate will flow back to the infusion bottle from the superior vena cava end of the liver after flowing through the liver.

The NMP device includes: blood transfusion pump, thermostatic water bath, small animal membrane oxygenator, filter, infusion connecting pipe, three-way valve, oxygen cylinder, flow pressure sensor (Figure 1).

## Methods

### Surgical procedures

The rats fasted 12 h before the experiment and could not refrain from water. Before laparotomy, the rats were weighed and anesthetized (2% pentobarbital sodium, 40 mg/kg for rat), and then fixed to the bench in supine position. Using scissors along the xiphoid process to the pubic bone opening in the midline, from the bladder above the incision to the left and right sides of the oblique upward until below the ribs, fixed, the abdominal cavity is fully exposed to view. A sterile and moist cotton swab was used to push the stomach, intestines and other organs or tissues of the rat to one side of the gauze, so that the liver was not covered and completely exposed to the field of vision. Sutures were applied to the proximal and distal ends of the inferior vena cava respectively and were treated during intubation. Renal vein and adrenal vein were layed, hepatic artery spare ring was layed, portal vein left and right branches were layed, and portal vein above the spare ring was layed. Ligate the distal hepatic end of the portal vein, intubate and fix it above the ligature. The hepatic artery and distal hepatic end of inferior vena cava were ligated, and the vessels above the distal hepatic end were cut. The liver was rinsed with a portal vein cannula. After rinsing, the inferior vena cava was lapped near the liver. Cut the septum and the ribs of the rat, and ligate the proximal end of the superior vena cava. A ring was inserted at the distal end, and an opening was made above it to intubate and fix it. Heparin was injected again through the portal vein to flush the liver, and the blood was drained through the superior vena cava. When the color gradually faded, the liver was removed and rinsed with normal saline, followed by immediate perfusion or preservation.

### Experimental groups

SD rats were randomized (*n* = 6) and the livers were perfused or preserved with the following solution.

Experiment 1 To explore the optimal addition ratio of pPolyHb in perfusion solution (37°C)

Group 1: Basal perfusate.

Group 2: 12% pPolyHb + basal perfusate.

Group 3: 24% pPolyHb + basal perfusate.

Group 4: 36% pPolyHb + basal perfusate.

Experiment 2 Evaluation of the effectiveness of the infusion solution with the best proportion of pPolyHb

Group 1: 24% pPolyHb + basal perfusate (37°C).

Group 2: Krebs Henseleit perfusate (37°C).

Group 3: UW solution (4°C).

### NMP process and sample collection

Open the water bath for heating in advance, set the temperature at 37°C, and input the gas composition as 95% O_2_ + 5% CO_2_. The liver is connected to the perfusion system and then open the transfusion pump and oxygenator. The perfusate flows into the liver through the superior vena cava and exits through the portal vein then circulates. After the beginning of perfusion, the perfusion fluid samples were collected from the portal vein at 0 h, 1 h, 3 h and 6 h, respectively.

### UW solution storage and sample collection

After liver removal, it was stored in UW solution at 4°C, and samples of the preservation solution were collected at 0 h, 1 h, 3 h, and 6 h.

### Assessment of liver function and injury

Detect the liver injury indicators alanine aminotransferase (ALT), aspartate aminotransferase (AST), lactate dehydrogenase (LDH), malonaldehyde (MDA)and monitor the changes of pH, pO2, pCO2, blood glucose (Glu), lactic acid (Lac) and other physiological indicators in real time. The pathological sections were stained with H&E staining and TUNEL staining to observe the morphological changes of liver tissue and the number of hepatocyte apoptosis. The expression of apoptosis protein Bcl-2 and Bax was detected by Western Blot.

### Statistical analysis

Data were statistically analyzed and plotted using Graphpad Prism 8.0. Each experimental group was repeated more than three times, statistical differences between groups were determined using One-Way ANOVA, and data were reported as mean ± SD. A p value less than 0.05 was considered statistically significant. Western Blot analysis detection data were analyzed using image J.

## Results

### Optimal addition ratio of pPolyHb

#### Liver function tests

**FIGURE 2:**
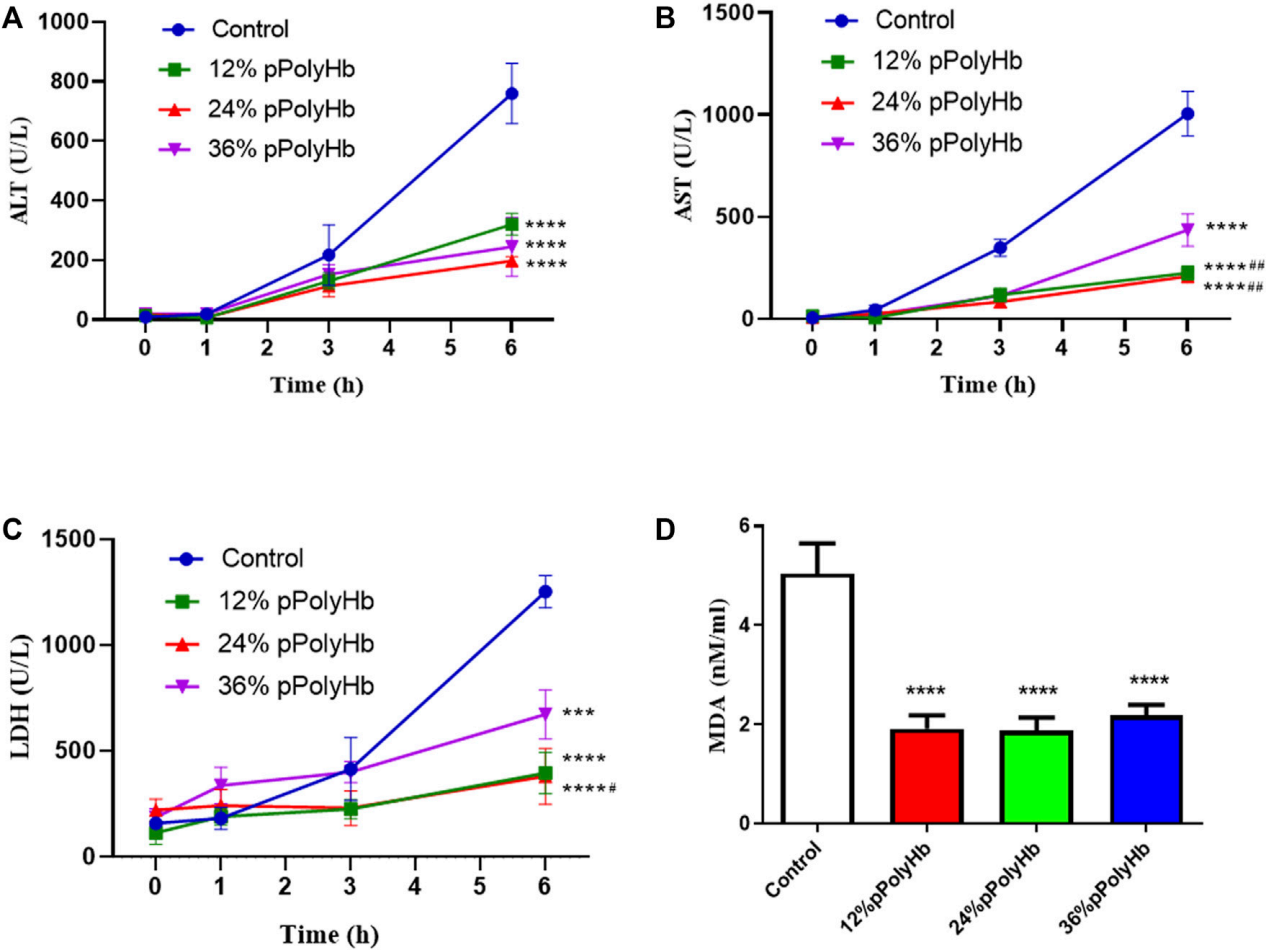
**(A)** After 1 h of perfusion, the levels of ALT in the three groups supplemented with pPolyHb were significantly lower than those in basal perfusate group (****p < 0.0001) and the lowest level was found in the 24%pPolyHb group **(B)** After 1 h of perfusion, AST levels in three groups supplemented with pPolyHb were significantly lower than those in basal perfusate group (****p < 0.0001) and the groups supplemented with 12% pPolyHb and 24% pPolyHb was significantly lower than 36% pPolyHb group (##*p* < 0.01) **(C)** LDH values of three groups supplemented with pPolyHb were lower than those of basal perfusate group (***p < 0.001, ****p < 0.0001). The 24% pPolyHb group was significantly lower than 36% pPolyHb (#*p* < 0.05) **(D)** MDA levels in basal perfusate group was significantly higher than that in pPolyHb group (****p < 0.0001) (Note: * stands for comparison with the control group, # stands for comparison with 36% pPolyHb, *p < 0.05. **p < 0.01, ***p < 0.001, ****p < 0.0001, # Similarly).

Enzyme activity. The release of hepatocyte enzymes (ALT, AST, LDH) in the three groups supplemented with 12% pPolyHb, 24% pPolyHb, and 36% pPolyHb was significantly lower than that in the basal perfusate group. Among the three groups supplemented with pPolyHb, the ALT level in the 24% pPolyHb group increased the most slowly. The AST values of 24% pPolyHb group and 36% pPolyHb group had the same upward trend and similar values, and the AST values of both groups remained at 0–300 (U/L). The most sensitive index of LDH activity increased as 36% pPolyHb group had the highest value, 12% pPolyHb group was close to 24% pPolyHb group. The tissue MDA production in basal perfusate group was significantly higher than that in the three groups supplemented with pPolyHb (Figure 2).

#### Blood gas analysis

**FIGURE 3:**
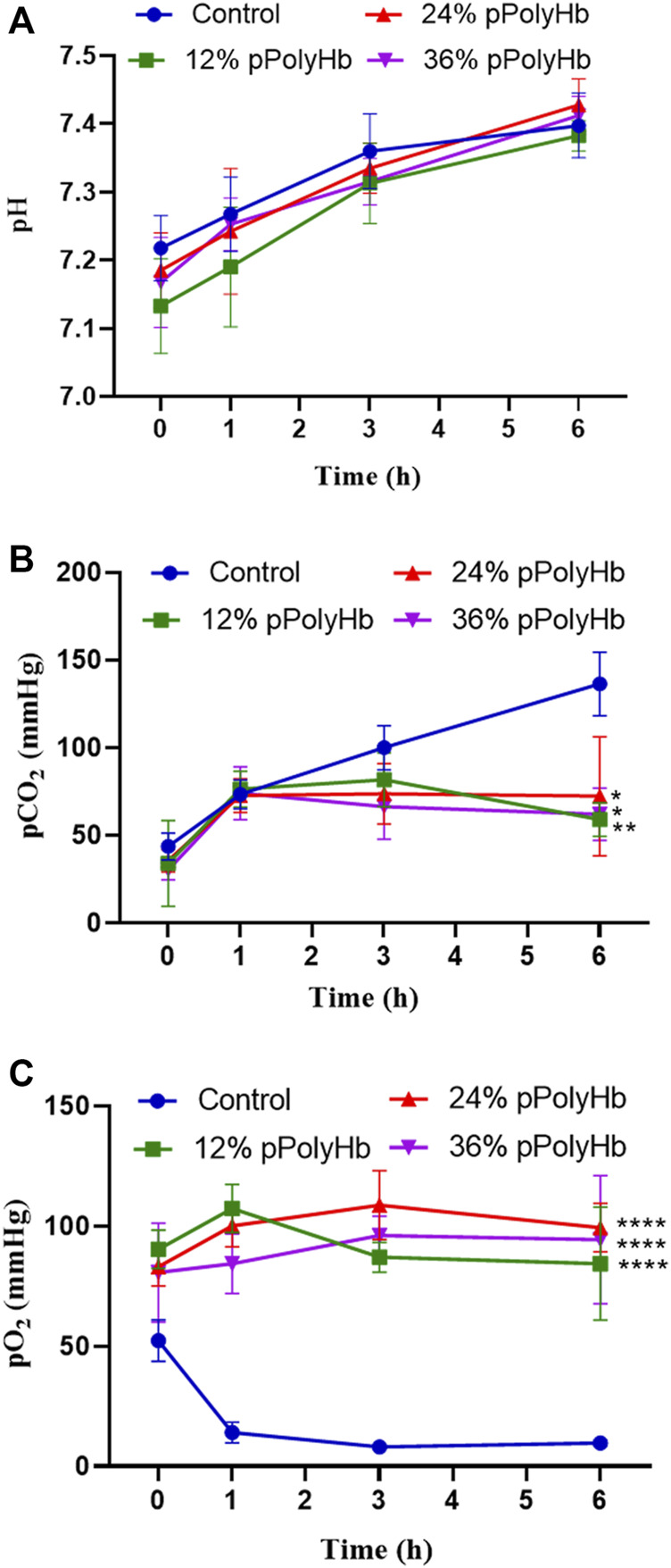
**(A)** There was no significant difference in pH between the four groups during perfusion **(B)** pCO2 in the three groups supplemented with pPolyHb was significantly lower than that in basal perfusate group (*p < 0.05, **p < 0.005) **(C)** pO2 in the three groups supplemented with pPolyHb during perfusion was significantly higher than that in basal perfusate group (****p < 0.0001) (Note: * stands for comparison with the control group).

pH. Within 6 h of perfusion, the pH of perfusate did not deviate from the normal range in all groups, but it increased slightly in all groups from the beginning to the end of perfusion (Figure 3A).

pCO_2_. At the beginning of perfusion, the pCO_2_ of four groups was lower than 50 mmHg, the pCO_2_ of the basal perfusate group increased to 130 mmHg with time. pCO_2_ increased from 0 h to 1 h in three groups supplemented with pPolyHb. pCO_2_ increased slightly from 1 h to 3 h in the 12% pPolyHb group, and then decreased slowly from 3 h to 6 h pCO_2_ in 24% pPolyHb group and 36%pPolyHb group tended to be stable after 1 h until the end of perfusion (Figure 3B).

pO_2_. Within 6 h, pO_2_ in the basal perfusate group decreased from 50 mmHg at the beginning to less than 10 mmHg at the end, while pO_2_ in three groups supplemented with pPolyHb was in the range of 83 ± 6.230 mmHg to 108.667 ± 14.364 mmHg (Figure 3C).

#### Electrolyte

**FIGURE 4:**
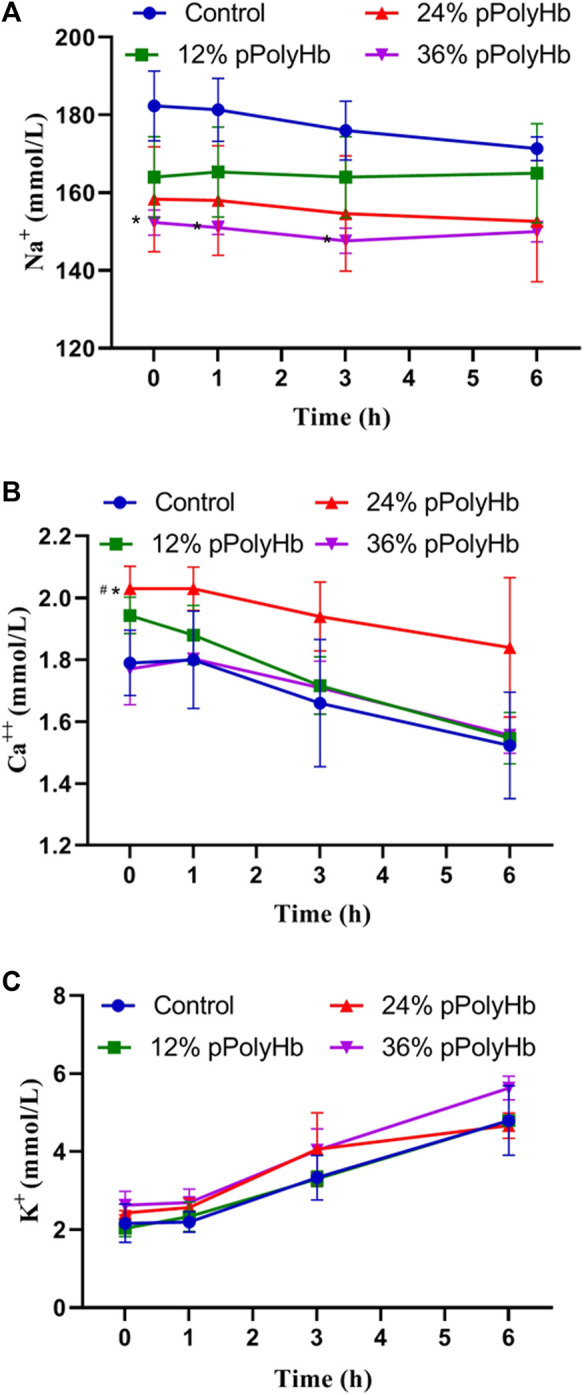
(A) There was no significant difference in Na + concentration among the four groups during perfusion (B) After 1 h of perfusion, Ca2+ in the four groups showed a downward trend, and after the end of perfusion, Ca2+ in the 24% pPolyHb group was slightly higher than that in the other three groups (C) There was no significant difference in K+ concentration among the four groups, and it increased at any time (Note: * stands for comparison with the control group, # stands for comparison with 36% pPolyHb, *p < 0.05, # Similarly).

During the perfusion period, the change range of Na^+^ was relatively stable, ranging from 147.67 ± 3.215 mmol/L to 182.75 ± 8.963 mmol/L, and the change range of K^+^ was from 2.03 ± 0.208 mmol/L to 5.63 ± 0.306 mmol/L. The basal perfusate group and three groups supplemented with pPolyHb showed the same trend of change, and the concentration increased with time. Ca^2+^ decreased regularly in the range of 1.523 ± 0.172 mmol/L to 2.03 ± 0.07 mmol/L, the highest concentration not exceeding the normal range (Figure 4).

#### Blood glucose and lactic acid

**FIGURE 5:**
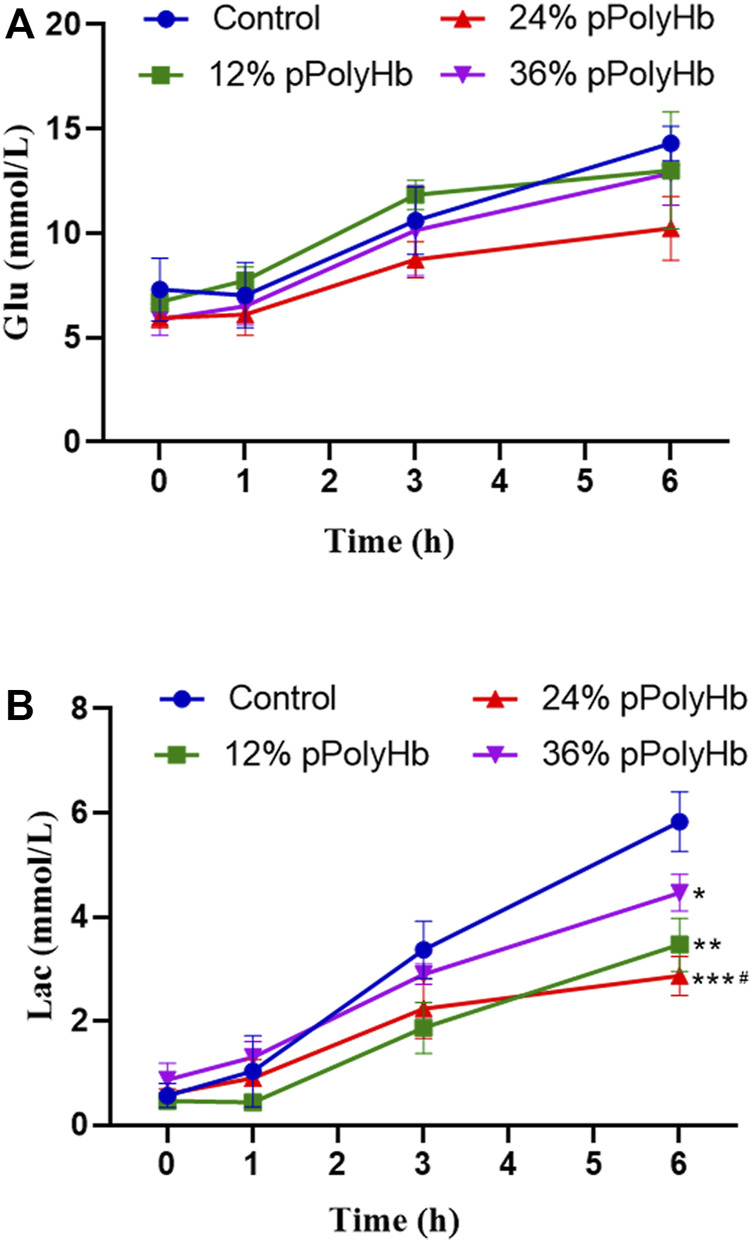
**(A)** The trend in glucose change for each group throughout perfusion. The blood glucose levels in all four perfusion groups rose as perfusion duration was extended, however the 24% pPolyHb group’s perfusion group’s blood glucose increase trend was the lowest **(B)** The amount of lactic acid created by each group during perfusion, with the control group producing the highest, the 36% pPolyHb group coming in second, and the 24% pPolyHb group coming in last (Note: * stands for comparison with the control group, # stands for comparison with 36% pPolyHb, *p < 0.05. **p < 0.01, ***p < 0.001, ****p < 0.0001, # Similarly).

Blood glucose. Blood glucose increased with perfusion time in all groups and overall changed in the range from 5.867 ± 0.751 mmol/L to 14.3 ± 0.819 mmol/L). There was no significant difference among four groups within 0 h–1 h, but four groups increased significantly within 1h–3 h. At 3 h–6 h, there was also an increase in each group, but the value of three groups supplemented with pPolyHb increased slowly compared with basal perfusate group (Figure 5A).

Lactic acid. With the prolongation of perfusion time, the lactic acid value of four groups gradually increased, and the overall change was in the range of 0.433 ± 0.115 mmol/L to 5.833 ± 0.569 mmol/L. The lactic acid value of the 24% pPolyHb group was 2.233 mmol/L at 3 h, and the other three groups supplemented with pPolyHb was lower than the 3-h threshold of 2.5 mmol/L recommended by Mergental (Mergental et al., 2020) et al. From 3 h to 6 h of perfusion, the lactate increase rate of three groups supplemented with pPolyHb was slowed down, and the most significant was the 24% pPolyHb group, and the lactic acid value at 6 h was still close to the threshold of 2.5 mmol/L. However, the lactic acid value of basal perfusate group continued rising, reaching 3.367 mmol/L at 3 h and 5.833 mmol/L at 6 h (Figure 5B).

#### Histology

**FIGURE 6:**
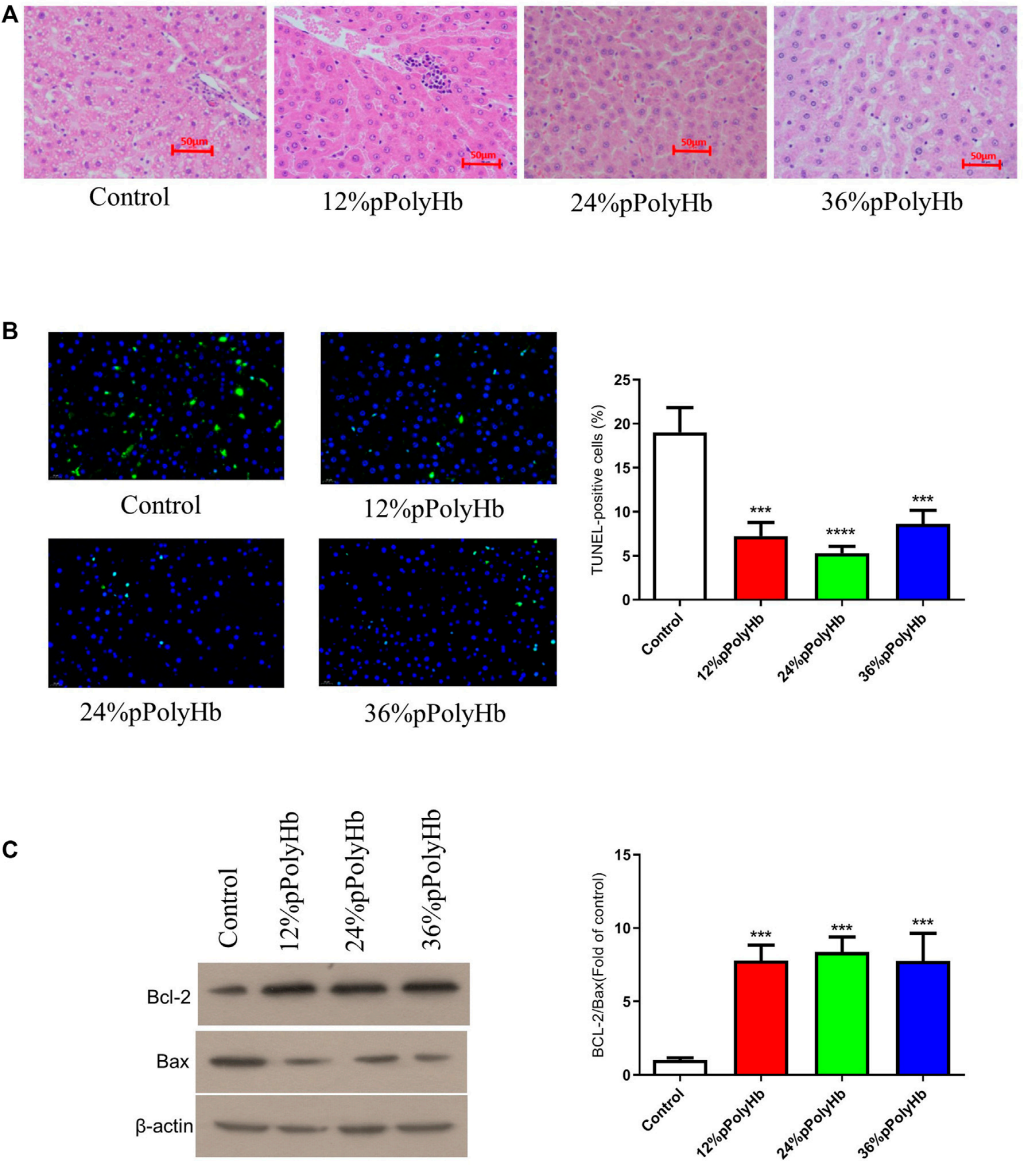
**(A)** H&E straining. Different groups show different physiological morphology, among which 24% pPolyHb group is the best **(B)** TUNEL straining. The control group has the largest number of apoptotic cells. The number of apoptotic cells in 12% pPolyHb group and 36% pPolyHb group is about half of that in control group. The number of apoptotic cells in 24% pPolyHb group was the lowest **(C)** Expression levels of Bax and Bcl-2 in hepatocytes of each group and the ratio of Bcl-2/Bax in each group.

H&E staining. H&E staining was performed on liver slices. Normal rat liver has clear nucleoplasm and nuclear plumpness, without vacuolar degeneration or necrosis. The staining of hepatocytes showed that the nuclei of the basal perfusate fluid group were shrunk, irregular in shape and appeared many vacuoles. 12% pPolyHb group showed clear hepatic lobules and hepatic cords, but multiple punctal necrosis of paravascular hepatocytes. In 24% pPolyHb group, the hepatocytes were arranged neatly, the boundary between nuclear and cytoplasm was clear, and the hepatic lobule structure was intact. 36% pPolyHb group images showed slight atrophy of liver cells (Figure 6A). In conclusion, compared with the basal perfusate group, the liver injury degree of three groups supplemented with pPolyHb after perfusion was reduced, and 24% pPolyHb group had the lowest injury degree.

TUNEL staining was performed on liver tissue sections after different treatments. The results showed that the number of apoptotic cells in basal perfusate group was the highest, and the number of apoptotic cells in 24% pPolyHb group was the lowest (Figure 6B). It was further indicated that the addition of pPolyHb could effectively protect the liver and the degree of liver injury was lower in 24% pPolyHb group.

#### The expression of apoptotic protein Bcl-2 and bax

The expression of apoptotic protein in liver tissue after perfusion with different pPolyHb content was detected by Western Blot. The results showed that compared with the basal perfusate group, the expression of Bcl-2 was higher and the expression of Bax was lower in the three groups supplemented with pPolyHb, indicating that the addition of pPolyHb in the perfusate had a protective effect. By comparing the values of Bcl-2/Bax, it can be seen that the protein apoptosis rate of the three groups supplemented with pPolyHb was significantly reduced, and 24% pPolyHb had the lowest apoptosis rate (Figure 6C).

### Comparison of basal perfusate + 24% pPolyHb with K-H solution and UW solution for liver protection

#### Liver function test

**FIGURE 7:**
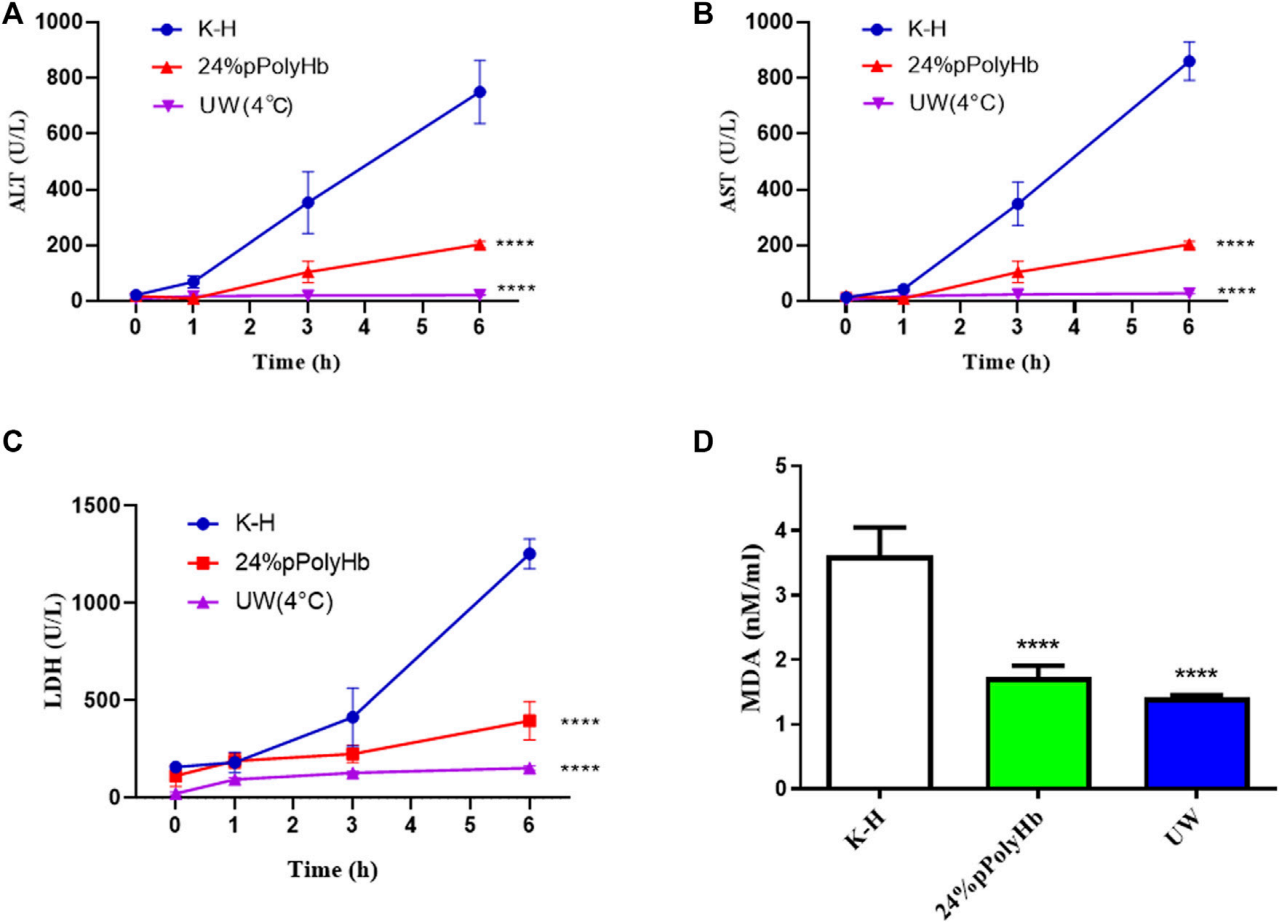
**(A)** Within 1 h after perfusion, ALT of K-H solution group was higher than the other two groups. One hour after perfusion, two groups were significantly lower than K-H solution group (* * * *p < 0.0001) **(B)** After 1 h of perfusion, AST of K-H solution group was significantly higher than the other two groups (* * * *p < 0.0001) **(C)** After 1 h of perfusion, LDH of K-H solution group was significantly higher than the other two groups (* * * *p < 0.0001) **(D)** After 1 h of perfusion, MDA of K-H solution group was significantly higher than that of the other two groups (* * * *p < 0.0001).

Enzyme activity. Among the three groups, the values of hepatocyte enzymes (ALT, AST, LDH) in UW solution and 24% pPolyHb group were significantly lower than those in K-H solution group. The amount of hepatocyte enzyme released in K-H solution group was significantly increased after 1 h perfusion. In the 24% pPolyHb group, ALT and AST values increased gradually from 0 h to 6 h, but maintained within the range of 0–200 U/L. LDH also increased gradually, and maintained in the range of 0–400 U/L from the beginning to the end of perfusion. In addition, the MDA value of 24% pPolyHb group and UW solution group was significantly lower than that of K-H solution group (Figure 7).

#### Histology

**FIGURE 8:**
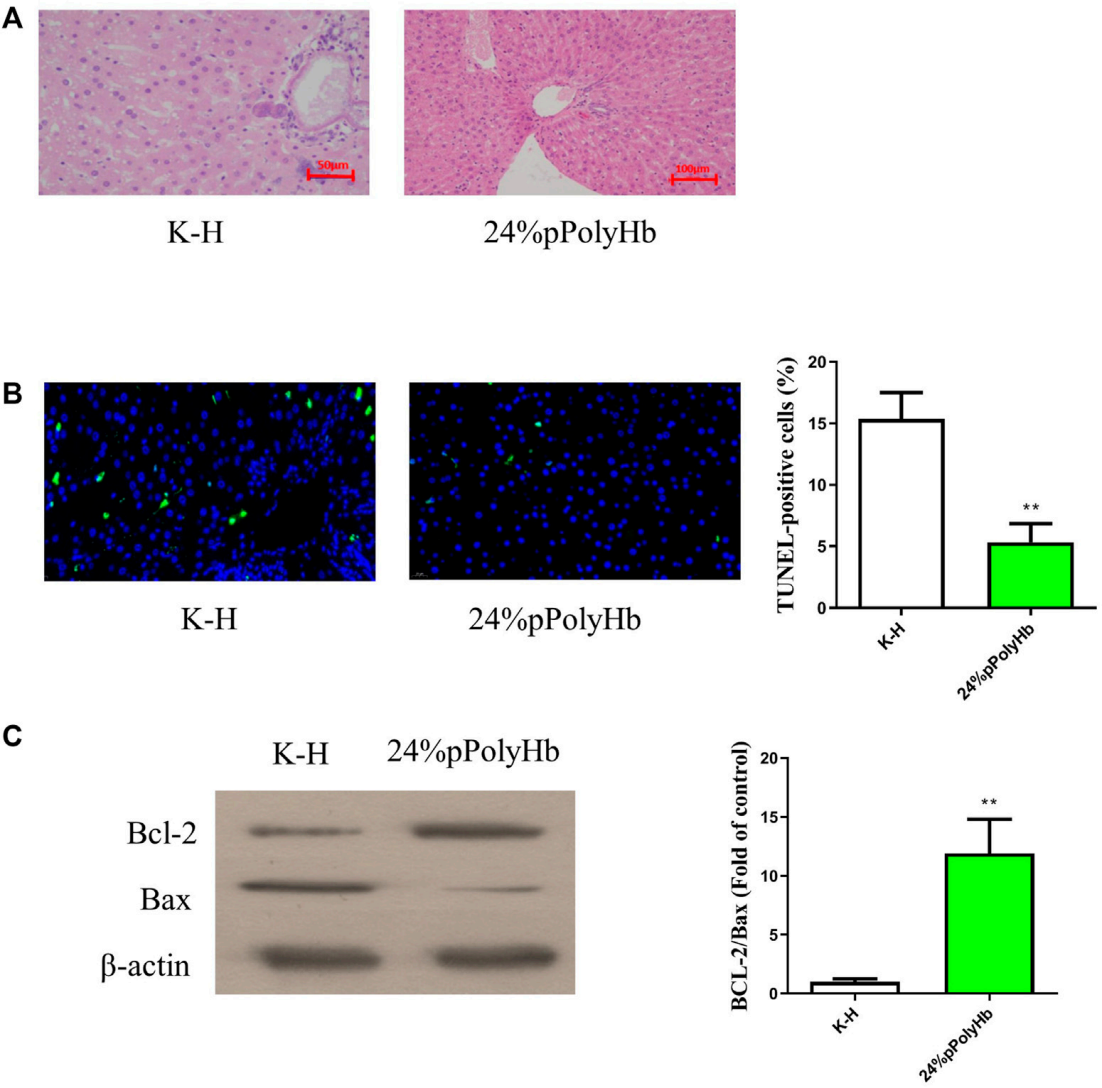
**(A)** H&E staining results. The liver tissue infused with K-H solution was highly vacuolated, and the structure of hepatic lobule was changed. After perfusion of 24% pPolyHb, the liver tissue and hepatic cord were clear, distinct nucleoplasm, and the nucleolus could be clearly observed **(B)** TUNEL staining results. The K-H solution group has a large amount of blue fluorescence. The number of apoptotic cells in K-H solution group was significantly higher than that in 24% pPolyHb group (* *p < 0.01) **(C)** Expression of apoptosis related proteins Bax and Bcl-2. The results of Western Blot showed that the expression level of Bcl-2 was higher in 24% pPolyHb group, and the expression level of Bax was lower. According to the histogram, the Bcl-2/Bax of 24% pPolyHb group is significantly higher than that of K-H solution group (* *p < 0.01).

The results of H&E staining showed that the hepatic lobules and cords of the liver tissue after perfusing with the 24% pPolyHb group was clear, distinct nucleoplasm, and the nucleolus could be clearly observed. However, the liver tissue perfused with K-H solution was highly vacuolated, and the hepatic lobule structure was changed (Figure 8A). TUNEL staining showed that the number of apoptotic cells in K-H solution group was significantly higher than that in the 24% pPolyHb group (Figure 8B).

#### Expression of apoptotic protein Bcl-2 and bax

The apoptotic protein expression levels after K-H solution and 24% pPolyHb perfusate treatment were detected by Western Blot. It was found that the expression of Bcl-2 after K-H solution infusion was lower than that after 24% pPolyHb perfusate infusion, and the expression of Bax was higher (Figure 8C).

## Discussion

Since the success of the first liver transplantation (LT), it has become a recognized treatment for patients with end-stage liver disease (Adam et al., 2012). Organ preservation is an important part of organ transplantation. The quality of the organ should be maintained during the interval between the removal from the donor and the transplantation into the recipient (Weissenbacher et al., 2019). At present, the most commonly method of organ preservation in clinic is SCS, which goal is to reduce the energy consumption of organs through static low temperature, so as to prolong the preservation time. Although SCS reduces the enzyme activity and oxygen consumption in organs and can maintain the intracellular ion homeostasis and membrane potential, the demand for energy metabolism and oxygen continues (Kasil et al., 2019). Anaerobic metabolism in SCS continues to cause the accumulation of metabolites, which leads to ischemia-reperfusion injury (IRI) after reperfusion of receptor organs (Chouchani et al., 2014). SCS is acceptable for high-quality organs, but it has limitation in high-risk or marginal organs. NMP allows organs to perform metabolism and liver function synthesis under physiological conditions, which theoretically provides the best condition for evaluating organ survival and integrity before transplantation (von Horn and Minor, 2019). David Nasralla et al. showed in a randomized trial of 220 liver transplants that compared with traditional static refrigeration, normothermic perfusion preservation can reduce graft damage by 50% (Nasralla et al., 2018). In an unprecedented feasibility reasearch, Eshmuminov et al. were able to perfuse the liver allograft for 7 days to keep the liver functional under normal temperature. The experimental results are far-reaching and widely cited, which show that long-term NMP is safe and feasible (Eshmuminov et al., 2020).

Additional oxygen supplementation during organ preservation can promote ATP synthesis and prevent anaerobic metabolism to reduce reperfusion injury (Minor et al., 2011). Nevertheless, direct oxidation may aggravate the oxidative stress damage of the organ if the oxidative metabolism of the graft is seriously damaged. Another method is to use oxygen carriers, especially those based on Hemoglobin-based oxygen carriers (HBOCs). HBOCs were originally studied as blood substitutes, but now they have been extended to the treatment of ischemia and hypoxia (Cao et al., 2021). Recently, the “repositioning” of HBOCs has led to its successful application in organ perfusion fluid, which is considered as an alternative method to improve oxygen supply and protect the optimal metabolic activity.

In this research, we used the hemoglobin oxygen carrier pPolyHb as a major component of perfusate for NMP of rat donor liver. In this paper, the effect of hyperkalemia caused by high concentration of potassium in perfusate on perfusion is not clear (Tan et al., 2005). The ideal composition of NMP perfusate is not yet clear, and most of the existing reports have differences in composition addition and functional differences in the regulation of various components. In this paper, we added some intravenous nutrient elements and trace elements to the perfusate to simulate the normal physiological environment of the liver during NMP and maintain the supply of nutrient elements. Although the mechanism of the necessity of these trace elements for short-term NMP has not been fully understood and confirmed, they may be necessary for long-term perfusion of isolated liver. Hydroxyethyl starch was used because it was isotonic and had a neutral pH, sodium and chlorine content were similar to the extracellular fluid, and salt components such as calcium chloride, potassium dihydrogen phosphate, magnesium sulfate, and potassium hydroxide were used to maintain electrolyte balance of the perfusate. Although the liver itself is expected to produce a certain amount of nutrients, we still add some exogenous nutrients such as glucose and compound amino acid buffer solution as appropriate supplement. On the basis of referring to Butler (García-Gil et al., 2011) basic perfusion formula, the perfusate in this paper has carried out a lot of optimization and improvement work, and the research results show that some data are slightly different from the expected, it demonstrated that our perfusate formula can be further optimized and upgraded in order to obtain better research results.

In hepatocytes, AST and ALT exist inside and outside mitochondria respectively. When liver is damaged, hepatocytes are destroyed, and the intracellular ALT and AST flow out of the cell, resulting in the increase of serum ALT and AST. LDH is widely distributed in the heart, kidney, liver and other sites, and its elevation can indicate myocardial infarction or hepatitis. In this experiment, normal saline was used to dilute the sample before detecting the perfusate, so as to exclude the possibility of false positive LDH. Some end points of our study showed that compared with liver perfusion without pPolyHb perfusate, the values of AST, ALT and LDH of damage markers were significantly decreased after the addition of pPolyHb. Although there is no clear indicator for the threshold of hepatocyte enzymes, the release of hepatocyte enzymes has important reference value for the evaluation of liver viability. In addition, as an oxygen carrier, the main function of pPolyHb is to better carry and release oxygen. In our comparison of partial pressure of oxygen between the group adding pPolyHb of different concentrations and the control group, it can be clearly found that the partial pressure of oxygen in the control group is extremely low, and from the overall curve trend, the partial pressure of oxygen in the 24% pPolyHb group is higher than 12% pPolyHb and 36% pPolyHb, indicating that the oxygen carrying and oxygen releasing capacity is better at this concentration.

Blood glucose is one of the important indicators to detect liver function. When liver function decreases, blood glucose will rise (Micó-Carnero et al., 2022). In this study, we added glucose to the perfusate to provide energy substrate for liver metabolism. In the follow-up test, it was found that the blood glucose increased in 1–3 h. Then it was discovered that the blood glucose of 12% pPolyHb group stopped rising in 3∼6 h, at the same time, the rising speed in 24% and 36% pPolyHb groups slowed down. The above results indicated that the liver was in the non-resuscitation state at the initial stage of perfusion, and the liver function declined by degrees. In the later period, the liver gradually recovered its metabolic function and the blood sugar was converted into glycogen then stored in the liver (Matton et al., 2019), thus reduced the measured blood sugar content. It showed that pPolyHb can protect the liver to some extent. Under the condition of hypoxia, blood glucose will undergo a large amount of anaerobic fermentation to convert into lactic acid (Weissenbacher et al., 2022). Lactic acid is an indicator of cell oxygen metabolism. In perfusion, its increase beyond the threshold indicates poor organ preservation effect, impaired liver function, and reduced lactate clearance rate (Aburawi et al., 2019). Previous research results showed that, from the beginning to the end of perfusion, although the lactic acid content in each group increased, the lactic acid value in the middle of perfusion was below the 3-h threshold of 2.5 mmol/L recommended by Mergental et al. At the end of perfusion, the lactic acid value of each group was still around this threshold. Among them, the lactate value of the perfusion group supplemented with 24% pPolyHb was the best, which indicated that the liver status improved after perfusion with 24% pPolyHb, and the lactate clearance ability was gradually restored. At the same time, low lactic acid value indicated that anaerobic metabolism in liver was improved, which further proved that pPolyHb had good oxygen carrying and oxygen releasing capacity.

The results of H&E staining and TUNEL staining after liver tissue sections were consistent: 24% pPolyHb group showed the best morphology of liver cells, and the number of apoptotic cells were the least. The outcomes showed that perfusion with this pPolyHb concentration had the best liver protection effect. Both Bax and Bcl-2 proteins belong to the Bcl-2 gene family, but their regulatory mechanisms are completely different (Tan et al., 2005). The three domains BH1, BH2 and BH3 in Bax gene can promote apoptosis. BH4 contained in Bcl-2 gene is a unique homologous domain of anti-apoptotic protein, which plays a role in inhibiting apoptosis (Sitailo et al., 2009). The comprehensive data of Western Blot analysis showed that the expression of Bax in 24% pPolyHb group was the lowest, and the Bcl-2 was the highest. This illustrated that pPolyHb can protect the liver by changing the expression of Bax and Bcl-2 proteins.

In addition, in the samples treated with pPolyHb perfusion solution, K-H perfusion solution and UW solution respectively, the levels of liver injury indicators in the K-H solution group were significantly higher than the other two groups in terms of ALT, AST, LDH or MDA. According to the results of H&E staining and TUNEL staining of 24% pPolyHb group and K-H solution group after normothermic machine perfusion, 24% pPolyHb group showed the best results in terms of the morphology of hepatic lobule and hepatic cord in liver tissue and the apoptotic cells. And from the ratio of Bcl-2/Bax, 24% pPolyHb group was significantly higher than K-H solution group, indicating that its addition could conspicuously increase the expression of anti-apoptotic genes and reduce the expression of pro-apoptotic genes. According to the above results, the effect after perfusion with oxygen carrier pPolyHb was obviously better than that after perfusion with K-H solution, which indicated that the addition of pPolyHb enhanced the ability of oxygen carrying and oxygen releasing to a certain extent, and reduced liver injury.

At present, NMP is a research hotspot in the field of organ preservation (Marecki et al., 2017; Nasralla et al., 2018; Bral et al., 2019). This technology has been proved to not only achieve the same preservation effect as cold preserved organs (op den Dries et al., 2016; Dutkowski et al., 2019), but also be able to repair marginal organs and damaged organs in some donor pools, and evaluate the physiological status of donor organs before transplantation. Our research demonstrates that pPolyHb can protect the liver to some extent and has explored the optimal addition ratio of 24%, but there are still some limitations: the number of experimental livers are still small; compared with experimental conditions with perfusion time of 10–24 h, our 6 h *in vitro* operation time is relatively short. In addition, this small animal NMP model does not currently include postperfusion transplantation. Therefore, it is not possible to evaluate liver function after transplantation.

## Conclusion

This study preliminarily explored the feasibility of pPolyHb as the main component of liver preservation solution, and proved that the isolated liver perfusion based on pPolyHb can basically maintain the physiological and biochemical functions of the liver within 6 h which confirmed that the pPolyHb based perfusion solution could be applied to the normal temperature machine perfusion of the isolated rat liver.

## Data Availability

The original contributions presented in the study are included in the article/Supplementary Material, further inquiries can be directed to the corresponding author.
